# Erosive and Mechanical Tooth Wear in Viking Age Icelanders

**DOI:** 10.3390/dj5030024

**Published:** 2017-08-29

**Authors:** Svend Richter, Sigfus Thor Eliasson

**Affiliations:** Faculty of Odontology, University of Iceland, IS101 Reykjavik, Iceland; sigfuse@hi.is

**Keywords:** enamel erosion, mechanical tooth wear, Icelandic settlers

## Abstract

(1) Background: The importance of the Icelandic Sagas as a source of information about diet habits in medieval Iceland, and possibly other Nordic countries, is obvious. Extensive tooth wear in archaeological material worldwide has revealed that the main cause of this wear is believed to have been a coarse diet. Near the volcano Hekla, 66 skeletons dated from before 1104 were excavated, and 49 skulls could be evaluated for tooth wear. The purpose of this study was to determine the main causes of tooth wear in light of diet and beverage consumption described in the Sagas; (2) Materials and methods: Two methods were used to evaluate tooth wear and seven for age estimation; (3) Results: Extensive tooth wear was seen in all of the groups, increasing with age. The first molars had the highest score, with no difference between sexes. These had all the similarities seen in wear from a coarse diet, but also presented with characteristics that are seen in erosion in modern Icelanders, through consuming excessive amounts of soft drinks. According to the Sagas, acidic whey was a daily drink and was used for the preservation of food in Iceland, until fairly recently; (4) Conclusions: It is postulated that the consumption of acidic drinks and food, in addition to a coarse and rough diet, played a significant role in the dental wear seen in ancient Icelanders.

## 1. Introduction

Wear is a generic term used in dentistry to describe phenomena of attrition (proximal and occlusal inter-dental friction), abrasion (friction with the intervention of particles) and erosion (chemical dissolution). This terminology suggests that these three phenomena act independently, whereas in fact it is more often the case that they interact simultaneously, which makes the diagnosis more difficult [1]. Dental erosion is tooth surface loss caused by extrinsic or intrinsic forms of acid. Extrinsic erosion is due to a highly acidic diet, while intrinsic erosion is caused by regurgitation of gastric acids [2]. Erosion softens the dental hard tissues, making them more susceptible to attrition and abrasion. Thus, if erosion and bruxism both exist, surface loss due to attrition is faster. The different physiological processes of tooth wear (abrasion, attrition and erosion) usually occur simultaneously and rarely work individually. Therefore, it is important to understand these tooth wear processes and their interactions to determine causes of tooth surface loss [1].

It is the norm to see extensive tooth wear in all ancient societies and is considered a major cause of tooth problems [1]. Tooth wear studies based on skull material from archaeological excavations have mainly been attributed to factors related to diet [3,4]. Acid erosion is hardly ever mentioned as a possible cause of this observed tooth wear [2].

From skeletal remains, evidence for dental health of ancient populations can be provided. The study of the ancient dentition can assist in reconstructing the lifestyle patterns and shed light on dental and general health. Among the conditions considered are ante mortem tooth loss, root abscesses, caries frequency, alveolar bone loss and occlusal tooth wear [5]. Hypo-mineralization of enamel, enamel hypoplasia, appearing as lines or pits, particularly on incisors, can be an indication of malnutrition or infections during tooth formation [6].

Observation of dental wear patterns is one of the most commonly utilized methods of age estimation in adult skeletal remains in archaeology. The popularity of this method arises from the high survival rate of teeth within archaeological contexts and the relative ease with which dental wear patterns can be observed and scored [7].

Research by trained dental personnel on dental health in ancient Icelanders has been almost nonexistent. Archaeologists and anthropologists have, however, noticed, registered and commented on excessive tooth wear without classifying or categorizing the type of wear [4,5].

In the years 1931–1939, archaeological and anthropological research was undertaken at the Skeljastadir farm in Thjorsardalur in Iceland. Sixty-six skeletons were excavated from an ancient graveyard: 5 infants, 2 children and 59 adults [8,9]. Volcanic ash from the mountain Hekla, dated from 1104, covered the skeletons [10]. The skeletons are preserved in the National Museum of Iceland.

The purpose of this investigation was to undertake detailed and thorough odontological investigation on the skulls to cast light on dental health in Viking Age Icelanders, especially in terms of the prevalence and type of tooth wear. 

## 2. Materials and Methods

The skeletons were examined at the stores of The National Museum of Iceland and are in relatively good condition. Of the 66 skeletons, 51 skulls could be used for the general dental health investigation and 49 skulls with 915 teeth could be evaluated for tooth wear.

The examination of the skulls and teeth was performed blinded by the two authors. Agreement was on more than 90% of all measurements. When there was a disagreement, an agreement was reached based on re-examination and discussion. 

**Figure 1:**
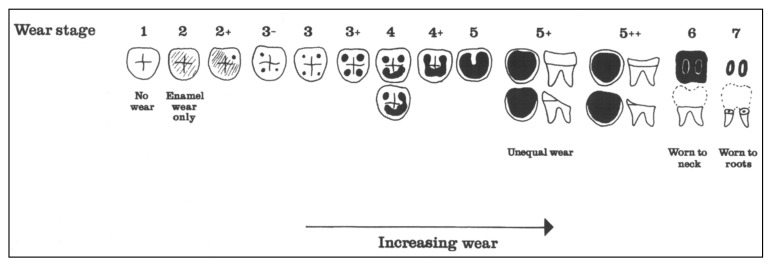
Brothwell classification of molar wear. White denotes enamel, black is exposed dentine [22].

Seven methods were used for age estimation, five were based on tooth development [11,12,13,14,15], one on tooth wear [16] and one on ectodermal suture closure [17]. Gender analysis was performed by using morphological characteristics from the skull, mandible and in a few instances, pelvis [5,18,19,20,21]. Two methods were used to register tooth wear. The first method was simply to register the wear into four categories: (1) no wear, (2) wear in enamel, (3) dentin exposed and (4) exposure of the pulp cavity. The second method was that developed by Brothwell, where wear was registered into 13 groups [22] as shown in Figure 1. The Brothwell method was limited to the skeletons of subjects 18 years and older: 22 males, 21 females and one where gender could not be determined. For statistical calculations, the scale for the wear stages was modified and numbered from 0 to 12 as shown in Table 1.

Tooth wear examination was performed with a dental explorer under good lighting and loupes with 2.8× magnification (Exam Vision ApS, Samsø, Denmark) and photographs with high resolution (Lester A. Dine Inc., Olympus Digital Camera Model No C-5060, Palm Beach Gardens, FL, USA). Conventional dental and radiographic examinations were performed for analyzing abscesses. A portable X-ray unit for X-ray analysis was used (Sirona, Helio Dent, Bensheim, Germany), as well as digital X-ray technology (Trophy RVG Digital X-ray Systems, Eastman Kodak Company, Rochester, NY, USA). Statistical analyses were made in the Stat View statistical software package (Stat View Software, SAS, Cary, NC, USA). R (The R Foundation for Statistical Computing, Vienna, Austria), RStudio (Rstudio, Boston, MA, USA) and Microsoft Excel (Microsoft Corporation, Redmont, WA, USA) were used for analyzing tooth wear in quartiles and the percentage of the scores, while other statistics were analyzed in Stat View.

## 3. Results

Gender distribution was equal: 24 females, 24 males and 1 of unknown sex. Tooth wear for each tooth number is shown in Table 2, where 0 means no detectable wear, 1 is wear in enamel, 2 is wear exposing dentin and 3 is wear exposing the pulp cavity. 

**Figure 2:**
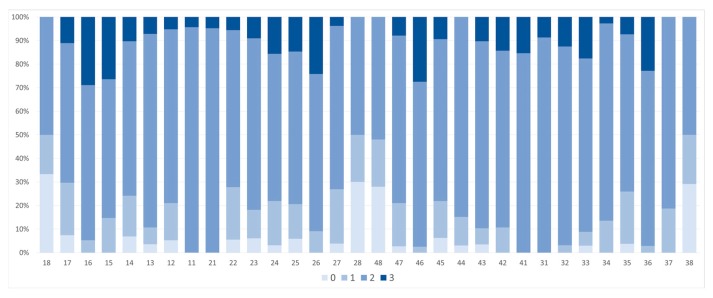
Distribution of tooth wear for each tooth number according to the classification: 0 no wear, 1 wear in enamel, 2 dentin exposed and 3 exposure of pulp cavity.

**Figure 3:**
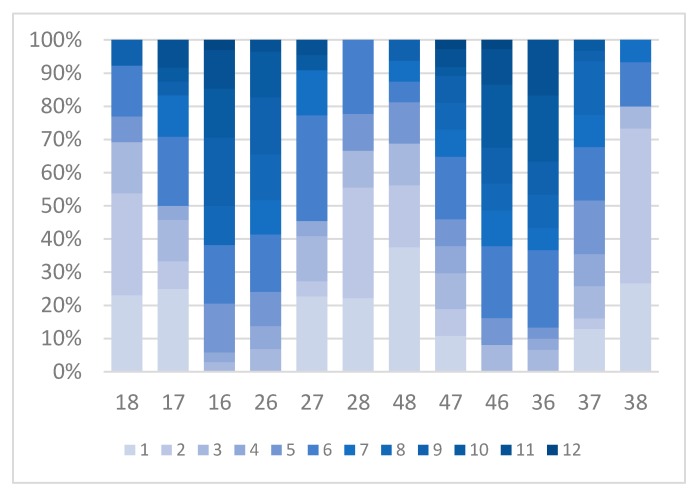
Distribution of tooth wear according to Brothwell, 18 years and older.

Distribution of tooth wear for each tooth number according to the classification: 0 no wear, 1 wear in enamel, 2 dentin exposed and 3 exposure of pulp cavity, can be seen in Figure 2 and in Figure 3, which shows the distribution of tooth wear according to Brothwell, 18 years and older. Tooth wear according to age and gender, using the Brothwell method, is shown in Table 3, Table 4 and Table 5, where tooth wear was only measured in skulls of subjects classified as being aged 18 years and older: 21 males, 21 females and one skull that where gender could not be determined. Tooth wear was higher in the age group 36 and older than for the 18–35 years group (Table 4), but no statistical difference between genders was noted (Table 5). Tooth wear with cuppings was registered in most molars in the younger age group and in the older age group where enamel was left (Figures 7 and 8). Root abscesses were found in 22 skulls out of 49, or in 45% of the cases. The abscesses were more common in the 36 years and older group, and significantly more common in males than females (Table 6) and the first molar showed the highest rate of abscesses (Table 7). Caries was almost non-existent, being registered in just two teeth.

## 4. Discussion

Previous studies of tooth wear have used clinical scoring systems [23,24,25,26] similar to this study. There is no universally accepted scoring system in the literature [1] and as a result, the use of different clinical indices prevents the direct comparison of different populations [1]. Generally, these clinical indices are quick, simple, and convenient with good reliability, since they usually have well-defined criteria with careful calibration amongst examiners [27,28,29].

**Figure 4:**
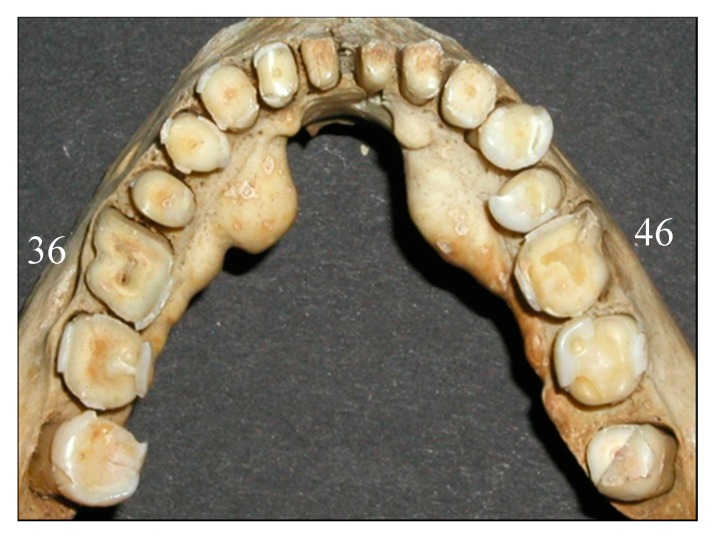
Secondary dentin formation seen in tooth 46, while tooth 36 has excessive wear with pulp exposure.

Tooth wear is seen in archaeological material from all over the world. This wear is generally far more extensive than can be seen in current living populations. Ancient people consumed a more course, rough and unprocessed diet, resulting in extensive occlusal tooth wear. This wear has been shown to gradually increase with age [3,5]. Usually the wear was most dominating on molars, starting in occlusal enamel and gradually reaching into dentin. Even though the wear reached well into the dentin, the teeth appeared to remain functional, since the odontoblastic activity prevented the wear from reaching into the pulpal cavity because of secondary dentin formation [22] (Figure 4).

The amount of wear can by graded by several methods. The simplest scoring of tooth wear is using only four categories: no wear, wear in enamel, wear into dentin and wear exposing the pulp cavity. When more detailed grading is preferred, several systems are available. The Brothwell method was elected to be used in the present study [22,30] (Figure 1). Tooth wear of each tooth type can be seen in Table 2. The third molars have the least wear and the first molars the most, with the second molars in between.

**Figure 5:**
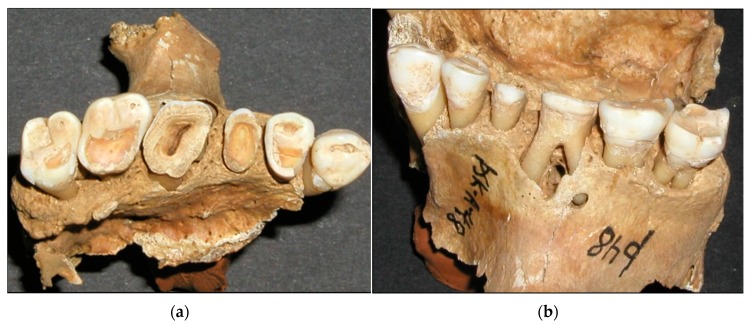
(**a**) Increasing tooth wear according to time of eruption, with pulp exposure in tooth 16. (**b**) Root abscess in tooth 16 due to tooth wear.

The amount of wear of the first molar indicates six years of wear when the second molar erupts. When the third molar erupts, the first molar has approximately twelve years of wear, and the second molar, six years of wear. Figure 5a,b, shows worn dentition from two angles. Different eruption times for the molars are, therefore, the basis for the age estimation. If, for example, the second molar shows that it has been in the mouth for 12 years and the third for six years, it is likely that the individual was about 24 years old. If the second molar shows 18 years of wear, it is likely that the individual was around 30 years old. Most of adulthood can be determined in the same way. The study by Miles indicated small differences in the wear rate between the three molars. He found the first molars to wear fastest, with a decreasing rate in the posterior direction. Thus, he showed that it took the second molars 6.5 years, and the third molars 7 years, to wear the same amount as the first molars in 6 years [30]. These wear differences are seen in Figure 5, explaining the age estimation method by Miles. Some studies have reported similar wear differentials [31] but others have reported equal rates of wear [32]. The Miles method was one of the methods used for age estimation in the present study. Many investigators have confirmed the usefulness of tooth wear for age estimation [32,33,34].

**Figure 6:**
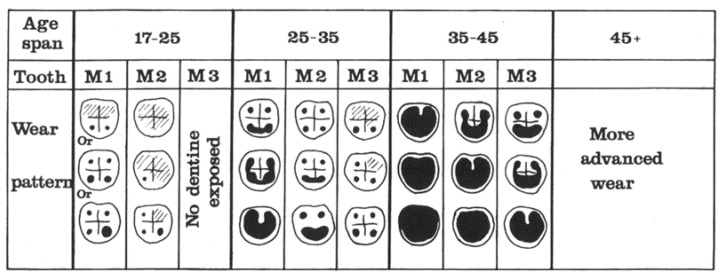
Estimated correspondence between adult age at death and molar wear phases for British material from Neolithic to medieval periods [35].

Brothwell used material from many British archaeological investigations when he examined the teeth of young people and found that tooth wear had changed little from Neolithic times (4000 B.C.) to the late middle ages (16th century). These findings helped him to develop a chart estimating tooth wear and age at death for these populations [22,30] (Figure 6). 

Tooth wear that is so excessive that it opens into the pulp cavity, as well as the effects on tooth physiology and pathology, need to be considered. Tooth wear is the cumulative loss of enamel and dentin and it is considered natural that teeth wear throughout life. Usually, the odontoblasts compensate for this wear with new, reactive, secondary dentin being laid down inside the pulp cavity, making the teeth functional and healthy [30]. Therefore, many investigators have used secondary or tertiary dentin as a foundation for developing methods for age estimation. These methods are based on radiographic measurements of the formation of new dentin and the shrinkage of the pulp cavity [15,36].

Several teeth in this investigation had wear exposure into the pulp cavity. Jon Steffensen, who excavated and originally investigated this bone material, found only one definite case where bone changes indicated scurvy or more likely vitamin D deficiency [3], although scurvy was known to be a common disease in Iceland until the 19th century [4,37]. Pindborg, supported by other authors, stated that scurvy could harm the odontoblasts, leading to irregular dentin formation that possibly wears at a faster rate [38]. Furthermore, Brothwell stated that in some instances, tooth wear could be so much and so fast that the normal odontoblastic activity is not enough to compensate for the wear, thereby resulting in an exposure into the pulp cavity [22]. Excessively worn dentition is shown in Figure 4, where tooth number 36 has an exposure into the pulp cavity, while visible secondary dentin formation has still compensated for the wear of tooth number 46. It is doubtful that vitamin C or D deficiency has resulted in exposure in only one first molar, although uneven wear is a possibility. 

Root abscesses were found in almost half of the skulls. Since the caries rate was very low for the population, it cannot explain the high root abscess incidence. A likely explanation is that excessive wear in some instances reached into the pulp cavity. This explanation is supported by the fact that first molars, which showed the highest score of tooth wear (Table 2), also had the greatest frequency of root abscesses (Table 7).

**Figure 7:**
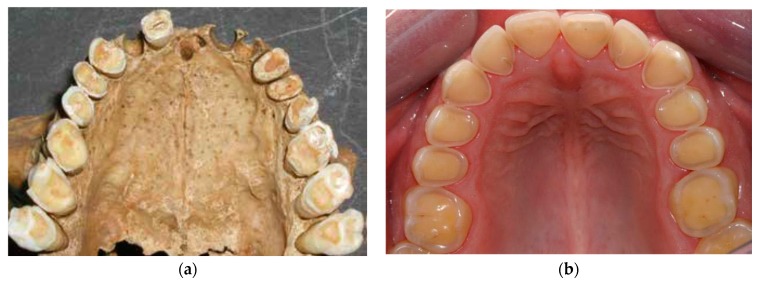
Similar characteristics (**a**) as seen in erosion in young people today (**b**).

The processed soft Western diet of today usually produces minor tooth wear. Recently, however, enamel erosion caused by excessive consumption of acidic beverages, mainly soft drinks, fruit juices and sport drinks, has become an epidemic among young people in Iceland [39], Figure 7.

**Figure 8:**
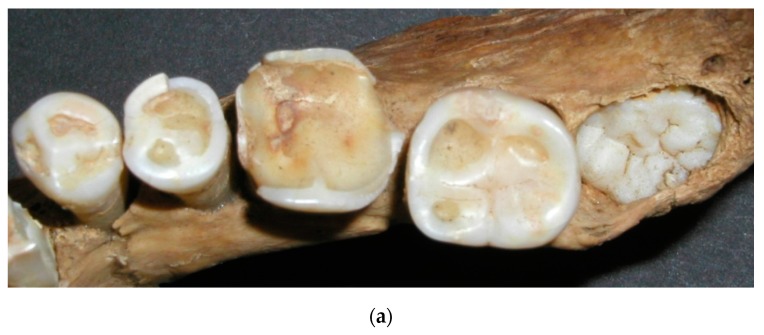

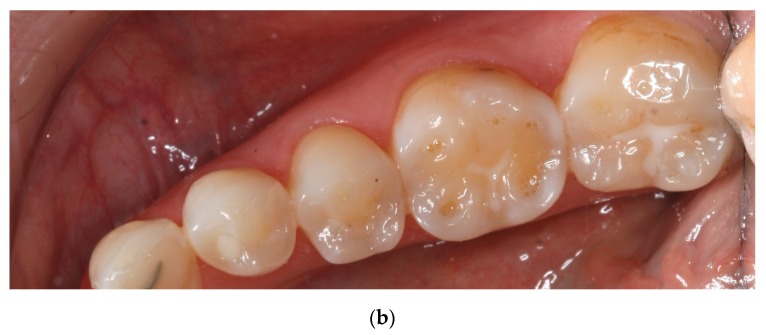
Similar appearance between the wear of medieval teeth (**a**) and the erosion with occlusal cupping in young people today (**b**).

Many teeth in the present study showed similar wear patterns to those seen in young people today that suffer from dental erosion. Figure 7 and Figure 8 show striking similarities between tooth wear in medieval teeth and in young people today with occlusal cupping. Cupping on tooth cusps and fissures is used as a criterion to discriminate lesions on enamel and dentin caused by dental erosion from abrasion/attrition worn flat wear facets [3,4,40].

Figure 8 shows clearly that the buccal and lingual enamel surfaces show little or no tooth wear. This suggests that the observed tooth wear was not caused by gastric reflux, even though lifting and other heavy work might have caused problems such as hiatus hernia, which could lead to gastric acid reaching the mouth and causing this typical pattern of tooth erosion. Although tooth erosion associated with gastric reflux disease is now quite common in Iceland, and has a typical clinical appearance, the prevalence of this in the medieval period is not known [41]. 

**Figure 9:**
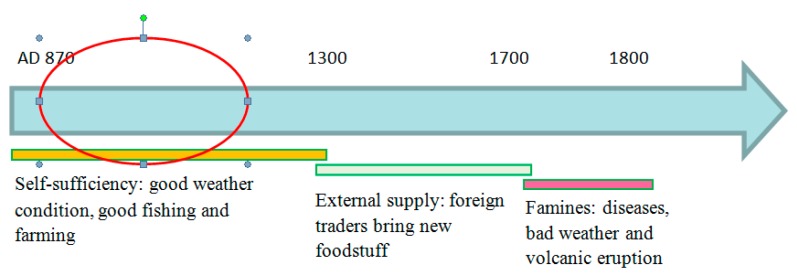
Timeline of Icelandic food history (adapted from Mehler 2011 [42]).

The history of food and nutrition in Iceland can shed light on tooth wear in ancient Icelanders. In Figure 9, a timeline of Icelandic food history can be seen [42]. Because of the lack of salt, drying was one of the two most popular ways for preserving food, mostly used on fish that was air-dried and cured as stock fish [43]. Meat was also dried, but it was more commonly soured in lactic acid. Due to the lack of grain, there are many tales in the literature about the dietary peculiarities of the Icelanders, which include the eating of dry fish instead of bread [43]. It is more than likely that in addition to the coarse diet, dried foodstuffs contaminated by dust, and in Thjorsardalur, volcanic ash too, were responsible for the extensive tooth wear in the medieval Icelanders. Furthermore, the lack of grain and fruits could be the explanation for the low caries rate in the skull samples.

Two dairy products are most characteristic of Icelandic foodstuffs. The first, namely whey (Icel. mysa), is a watery part of milk that separates from the curd in the process of making the second major diary product, skyr. Most of the whey was processed by pouring it into a wooden barrel with open holes in the lid for fermentation. When this process was finished, mysa had turned into lactic acid, sýra, which was then mixed with water in the ratio of one part acid with 11 parts water. This drink was the everyday thirst-quencher in Iceland until the mid-20th century [42]. Lactose is a disaccharide sugar found in milk and makes up around 2%–8% (by weight) of milk. As the whey fermented, about half of the lactose was converted to lactic acid along with other by-products [44]. Lactic acid was the most important food conservation medium in Iceland. Leathery meat was cooked and put into lactic acid afterwards to make it softer [42]. According to Saga literature, skyr was used in all Nordic countries during the middle Ages. In Iceland, skyr was eaten two or three times a day up until the 20th century [43]. 

Extensive studies have been made on tooth erosion in young people in Iceland in recent years. Dr. Thorbjorg Jensdottir has been at the forefront of those who have studied the erosive effects of acidic beverages in the country. The capability of acidic beverages to erode dental enamel depends not only on the pH of the drink, but also on its buffering capacity. Her study showed that the erosive potential of whey, mysa, was very high [45]. Mysa and Sýra in the medieval period is believed to have contained much higher concentrations of lactic acid than that which can be found in the same modern products, manufactured under much different and more controlled conditions [44].

**Figure 10:**
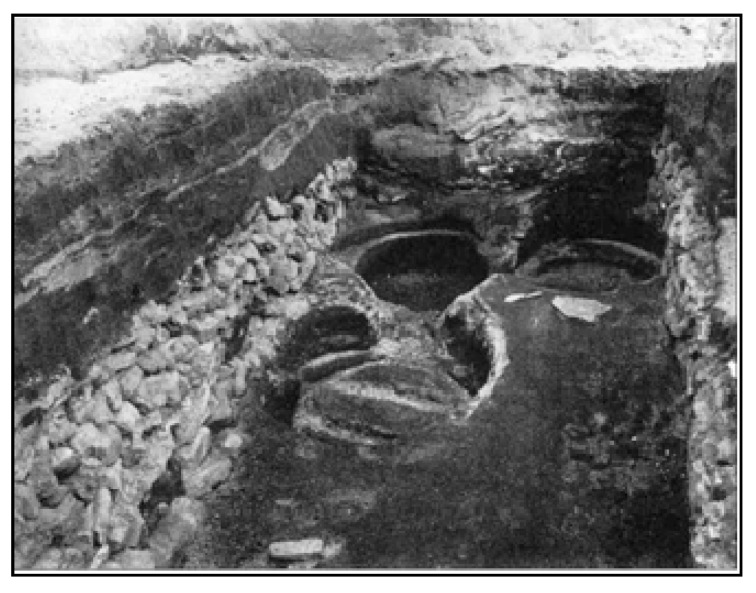
Whey barrel imprints in the storage room of the farm Stong (from Agustsson 1989 [46]).

In the Viking period, the storage of food or the production of dairy products commonly took place in small outhouses next to the longhouse. Up until the 20th century, Icelandic farms always included a room for the production of whey and skyr, and large wooden barrels or storage trunks were typical features of storage rooms, as seen in Figure 10, taken during the excavation of the Viking Age farm Stong in Thorsardalur, not far from the archaeological site, Skeljastadir [46].

## 5. Conclusions

In addition to the coarse diet of dried meat and fish, probably contaminated by abrasive material, it is postulated that acidic beverages and acidic food, were also responsible for the extensive tooth wear in medieval Icelanders.

## Figures and Tables

**Table 1:** Modification of Brothwell tooth wear.

	Scales for Wear Stages												
Brothwell Wear Stages (1981)	1	2	2+	3−	3	3+	4	4+	4	5+	5++	6	7
Brothwell Wear Stages (authors modification)	0	1	2	3	4	5	6	7	8	9	10	11	12

**Table 2:** Tooth wear for each tooth number according to the classification: 0 no wear, 1 wear in enamel, 2 dentin exposed and 3 exposure of pulp cavity.

Tooth Wear																
915 Teeth in 49 Skeletons																
Number of teeth	18	27	37	33	28	28	19	23	21	18	33	31	33	32	26	20
Highest value	2	3	3	3	3	3	3	3	3	3	3	3	3	3	2	2
Lowest value	0	0	1	1	0	0	0	2	2	0	0	0	0	1	0	0
75%	2	2	3	3	3	3	3	3	3	3	3	3	3	3	3	3
Median	2	2	2	2	2	2	2	2	2	2	2	2	2	2	2	2
25%	0	1	2	2	2	2	2	2	2	1	2	2	2	2	1	0
Tooth	18	17	16	15	14	13	12	11	21	22	23	24	25	26	27	28
Tooth	48	47	46	45	44	43	42	41	31	32	33	34	35	36	37	38
25%	0	2	2	2	2	2	2	2	2	2	2	2	2	2	2	0
Median	2	2	2	2	2	2	2	2	2	2	2	2	2	2	2	2
75%	2	2	3	2	2	2	2	2	2	2	2	2	2	2	2	2
Lowest value	0	0	1	0	0	0	1	2	2	1	0	1	0	1	1	0
Highest value	2	3	3	3	2	3	3	3	3	3	3	2	3	3	2	2
Number of teeth	25	38	39	31	32	28	28	25	23	32	34	37	26	34	32	24

**Table 3:** Tooth wear according to Brothwell modification, 18 years and older.

Brothwell, 18 Years and Older						
337 Teeth in 44 Skeletons						
Number of teeth	19	26	36	31	24	16
Highest value	9	11	12	11	11	6
Lowest value	0	1	3	3	1	0
75%	3	7	10	9	6	3
Median	2	5	9	7	6	2
25%	1	2	6	6	2	0
Tooth	18	17	16	26	27	28
Tooth	48	47	46	36	37	38
25%	1	3	6	6	4	1
Median	1	6	8	8	5	2
75%	3	8	10	10	7	2
Lowest value	0	1	3	1	1	0
Highest value	9	12	12	11	10	7
Number of teeth	23	38	39	32	32	21

**Table 4:** Tooth wear in 18–35 years and 36 years and older according to Brothwell.

Age Groups	Age 18–35						≥Age 36					
	121 Teeth in 13 Skeletons						207 Teeth in 31 Skeletons					
Number of teeth	7	9	9	11	10	7	11	16	26	19	13	8
Highest value	2	3	8	9	6	1	9	11	12	11	11	6
Lowest value	0	1	2	3	1	0	1	1	5	4	1	0
75%	1	2	6	7	4	1	6	8	10	10	7	5
Median	0	1	5	6	2	0	3	6	9	8	6	3
25%	0	1	4	4	1	0	2	6	8	7	6	2
Tooth	18	17	16	26	27	28	18	17	16	26	27	28
Tooth	48	47	46	36	37	38	48	47	46	36	37	38
25%	0	1	5	5	1	0	2	6	7	7	5	2
Median	1	3	6	6	3	0	4	7	9	10	6	2
75%	1	4	6	7	5	1	6	9	10	10	8	5
Lowest value	0	1	3	3	1	0	1	2	5	4	3	1
Highest value	3	5	8	9	5	2	9	12	12	11	10	7
Number of teeth	12	13	12	10	11	10	10	25	26	21	21	11

**Table 5:** Tooth wear for males and females according to Brothwell modification.

Gender	Males 18 Years and Older						Females 18 Years and Older					
	163 Teeth in 21 Skeletons						143 Teeth in 22 Skeletons					
Number of teeth	12	14	17	15	10	8	4	10	16	13	11	6
Highest value	9	11	12	10	11	6	5	10	11	11	7	6
Lowest value	0	1	3	3	1	2	0	1	4	4	1	0
75%	4	7	10	9	6	2	3	6	9	8	6	4
Median	2	6	9	7	5	2	2	4	7	6	6	1
25%	1	2	6	6	1	1	1	2	6	6	3	0
Tooth	18	17	16	26	27	28	18	17	16	26	27	28
Tooth	48	47	46	36	37	38	48	47	46	36	37	38
25%	0	3	5	7	3	1	1	4	6	6	4	0
Median	1	6	8	9	6	2	2	5	7	7	5	1
75%	5	7	10	10	8	4	3	9	10	10	7	2
Lowest value	0	1	3	3	1	0	0	1	5	4	1	0
Highest value	6	12	11	11	10	7	9	11	12	11	9	2
Number of teeth	9	18	17	15	16	12	11	18	19	14	14	7

**Table 6:** Prevalence of root abscesses according to age and gender.

Gender	Age	*n* Skeletons	*n* with Abscesses	% with Abscesses	% Abscesses
**Female**	35 years and below	11	2	18	36
	36 years and above	14	7	50	
**Male**	35 years and below	6	2	33	54
	36 years and above	18	11	61	
**Both Sexes**	35 years and below	17	4	24	45
	36 years and above	32	18	56	

**Table 7:** Number of root abscesses per tooth.

**Number of Abscesses**		1	9	3			2		1		1	1	1	5	1	
**Tooth**	**18**	**17**	**16**	**15**	**14**	**13**	**12**	**11**	**21**	**22**	**23**	**24**	**25**	**26**	**27**	**28**
**Tooth**	**48**	**47**	**46**	**45**	**44**	**43**	**42**	**41**	**31**	**32**	**33**	**34**	**35**	**36**	**37**	**38**
**Number of Abscesses**		1	6	1			1	2	1	1	3			5	1	

## References

[1] D’Incau E., Couture C., Maureille B. (2012). Human tooth wear in the past and the present: Tribological mechanisms, scoring systems, dental and skeletal compensations. Arch. Oral Biol..

[2] Johansson A. (1992). A cross-cultural study of occlusal tooth wear. Swed. Dent. J. Suppl..

[3] Steffensen J. (1943). Knoglene fra Skeljastaðir i Þjórsárdalur. Forntida Gårder i Island: Meddelanden Från Den Nordiska Arkeologiska Undersökningen i Island Sommaren 1939.

[4] Gestsdóttir H. (1998). The Palaeopathological Diagnosis of Nutritional Disease: A Study of the Skeletal Material from Skeljastaðir, Iceland. Master’s Thesis.

[5] Richter S. (2005). Odontological Investigation on Archaeological Human Remains from Skeljastadir in Thjorsardalur. Master’s Thesis.

[6] Caufield P.W., Li Y., Bromage T.G. (2012). Hypoplasia-associated severe early childhood caries—A proposed definition. J. Dent. Res..

[7] Millard A.R., Gowland R.L. (2002). A bayesian approach to the estimation of the age of humans from tooth development and wear. Archeol. Calcolatori.

[8] Þórðarson M. (1943). Skeljastaðir, þjórsárdalur. Forntida Gårder i Island: Meddelanden Från Den Nordiska Arkeologiska Undersökningen i Island Sommaren 1939.

[9] Steffensen J. (1943). Þjórsdælir hinir fornu. (flutt 14 desember 1941). Samtíð Og Saga: Nokkrir Háskólafyrirlestrar.

[10] Þórarinsson S. (1967). Beinagrindur og bókarspennsli. Árb. Hins Ísl. Fornleifafél..

[11] Demirjian A., Goldstein H. (1976). New systems for dental maturity based on seven and four teeth. Ann. Hum. Biol..

[12] Haavikko K. (1974). Tooth formation age estimated on a few selected teeth. A simple method for clinical use. Proc. Finn. Dent. Soc..

[13] Kullman L., Johanson G., Akesson L. (1992). Root development of the lower third molar and its relation to chronological age. Swed. Dent. J..

[14] Mincer H.H., Harris E.F., Berryman H.E. (1993). The A.B.F.O. Study of third molar development and its use as an estimator of chronological age. J. Forensic Sci..

[15] Kvaal S.I., Kolltveit K.M., Thomsen I.O., Solheim T. (1995). Age estimation of adults from dental radiographs. Forensic Sci. Int..

[16] Miles A.E.W. (1963). Dentition in the estimation of age. J. Dent. Res..

[17] Meindl R.S., Lovejoy C.O. (1985). Ectocranial suture closure: A revised method for the determination of skeletal age at death based on the lateral-anterior sutures. Am. J. Phys. Anthropol..

[18] Bass W. (1995). Human Osteology: A Loboratory and Field Manual of the Human Skeleton.

[19] Ubelaker D.H. (1989). Human Skeletal Remains: Excavation, Analysis, Interpretation.

[20] Duric M., Rakocevic Z., Donic D. (2005). The reliability of sex determination of skeletons from forensic context in the Balkans. Forensic Sci. Int..

[21] Mays S. (2003). The determination of age and sex. The archaeology of human bones.

[22] Ganss C. (2006). Definition of erosion and links to tooth wear. Monogr. Oral. Sci..

[23] Kreulen C.M., Van Spijker A., Rodriguez J.M., Bronkhorst E.M., Creugers N.H., Bartlett D.W. (2010). Systematic review of the prevalence of tooth wear in children and adolescents. Caries Res..

[24] Smith B.G., Bartlett D.W., Robb N.D. (1997). The prevalence, etiology and management of tooth wear in the United Kingdom. J. Prosthet. Dent..

[25] Normando D., Almeida M.A., Quintao C.C. (2013). Dental crowding: The role of genetics and tooth wear. Angle Orthod.

[26] Normando D., Faber J., Guerreiro J.F., Quintao C.C. (2011). Dental occlusion in a split amazon indigenous population: Genetics prevails over environment. PLoS ONE.

[27] Yun J.I., Lee J.Y., Chung J.W., Kho H.S., Kim Y.K. (2007). Age estimation of korean adults by occlusal tooth wear. J. Forensic Sci..

[28] Kullman L. (1995). Accuracy of two dental and one skeletal age estimation method in swedish adolescents. Forensic Sci. Int..

[29] Grimoud A.M., Roberts C.A., Boimond L., Sevin A., Lucas S., Passarrius O. (2012). Topographical presentation of dental wear as arches in a french mediaeval population. Arch. Oral Biol..

[30] Mays S. (2003). Redrawn from Miles (1963: Figure 10). The Archaeology of Human Bones.

[31] Miles A.E.W. (2001). The Miles method of assessing age from tooth wear revisited. J. Archaeol. Sci..

[32] Kieser J., Preston C., Evans W. (1983). Skeletal age at death: An evaluation of the Miles method of ageing. J. Archaeol. Sci..

[33] Tomenchuk J., Mayhall J.T. (1979). A correlation of tooth wear and age among modern igloolik eskimos. Am. J. Phys. Anthropol..

[34] Richards L.C., Miller S.L. (1991). Relationships between age and dental attrition in australian aboriginals. Am. J. Phys. Anthropol..

[35] Brothwell D. (1981). Dental attrition. Digging up Bones. The Excavation, Treatment and Study of Human Skeletal Remains.

[36] Paewinsky E., Pfeiffer H., Brinkmann B. (2005). Quantification of secondary dentine formation from orthopantomograms a contribution to forensic age estimation methods in adults. Int. J. Leg. Med..

[37] Johnsen B. (1968). Food in Iceland 874–1550. Medicinhist. Årsb..

[38] Pindborg J.J. (1965). De hårde tandvævs sygdomme.

[39] Arnadottir I.B., Saemundsson S.R., Holbrook W.P. (2003). Dental erosion in icelandic teenagers in relation to dietary and lifestyle factors. Acta Odontol. Scand..

[40] Khan F., Young W.G., Law V., Priest J., Daley T.J. (2001). Cupped lesions of early onset dental erosion in young Southeast Queensland adults. Aust. Dent. J..

[41] Holbrook W.P., Furuholm J., Gudmundsson K., Theodors A., Meurman J.H. (2009). Gastric reflux is a significant causative factor of tooth erosion. J. Dent. Res..

[42] Mehler N. (2011). From self-sufficiency to external supply and famine: Foodstuffs, their preparation and storage in Iceland. Processing, Storage, Distribution of Food: Food in the Medieval Rural Environment.

[43] Gísladóttir H. (1991). Eldhús og matur á Íslandi. Cand Mag Disertation.

[44] Lanigan L.T., Bartlett D.W. (2013). Tooth wear with an erosive component in a mediaeval Iceland population. Arch. Oral Biol..

[45] Jensdottir T., Thornorsdottir I., Arnadottir I.B., Holbrook W.P. (2002). Erosive drinks on the icelandic market. Laeknabladid.

[46] Ágústsson H., Líndal S. (1989). Húsagerð á síðmiðöldum. Saga Íslands. Bindi. IV.

